# A robust auto-contouring and data augmentation pipeline for adaptive MRI-guided radiotherapy of pancreatic cancer with a limited dataset

**DOI:** 10.1088/1361-6560/adabac

**Published:** 2025-01-30

**Authors:** Mehdi Shojaei, Björn Eiben, Jamie R McClelland, Simeon Nill, Alex Dunlop, Arabella Hunt, Brian Ng-Cheng-Hin, Uwe Oelfke

**Affiliations:** 1 Joint Department of Physics, Institute of Cancer Research and The Royal Marsden NHS Foundation Trust, London, United Kingdom; 2 The Royal Marsden NHS Foundation Trust, London, United Kingdom; 3 UCL Hawkes Institute and Department of Medical Physics and Biomedical Engineering, University College London, London, United Kingdom

**Keywords:** adaptive radiotherapy, pancreatic cancer, auto-segmentation, data augmentation, deep learning

## Abstract

*Objective.* This study aims to develop and evaluate a fast and robust deep learning-based auto-segmentation approach for organs at risk in MRI-guided radiotherapy of pancreatic cancer to overcome the problems of time-intensive manual contouring in online adaptive workflows. The research focuses on implementing novel data augmentation techniques to address the challenges posed by limited datasets. *Approach.* This study was conducted in two phases. In phase I, we selected and customized the best-performing segmentation model among ResU-Net, SegResNet, and nnU-Net, using 43 balanced 3DVane images from 10 patients with 5-fold cross-validation. Phase II focused on optimizing the chosen model through two advanced data augmentation approaches to improve performance and generalizability by increasing the effective input dataset: (1) a novel structure-guided deformation-based augmentation approach (sgDefAug) and (2) a generative adversarial network-based method using a cycleGAN (GANAug). These were compared with comprehensive conventional augmentations (ConvAug). The approaches were evaluated using geometric (Dice score, average surface distance (ASD)) and dosimetric (D2% and D50% from dose-volume histograms) criteria. *Main results.* The nnU-Net framework demonstrated superior performance (mean Dice: 0.78 ± 0.10, mean ASD: 3.92 ± 1.94 mm) compared to other models. The sgDefAug and GANAug approaches significantly improved model performance over ConvAug, with sgDefAug demonstrating slightly superior results (mean Dice: 0.84 ± 0.09, mean ASD: 3.14 ± 1.79 mm). The proposed methodology produced auto-contours in under 30 s, with 75% of organs showing less than 1% difference in D2% and D50% dose criteria compared to ground truth. *Significance.* The integration of the nnU-Net framework with our proposed novel augmentation technique effectively addresses the challenges of limited datasets and stringent time constraints in online adaptive radiotherapy for pancreatic cancer. Our approach offers a promising solution for streamlining online adaptive workflows and represents a substantial step forward in the practical application of auto-segmentation techniques in clinical radiotherapy settings.

## 1. Introduction

Radiation therapy (RT) can be used as a part of an overall treatment strategy for pancreatic cancer, particularly when surgical resection is not immediately feasible. However, delivering a sufficiently high radiation dose is challenging due to the proximity of critical organs highly sensitive to radiation-induced toxicity (Huguet *et al* 2009).

MR-Linacs, with high-quality imaging capabilities, enable clinicians to tailor treatment plans with greater accuracy, accommodating daily anatomical changes through adaptive planning strategies (Bohoudi *et al* 2017, Beets-Tan *et al* 2018). However, daily manual contouring of organs at risk (OARs) and the radiation target is a time-intensive process that can take hours to complete. This is impractical within the constraints of an online adaptive workflow. Therefore, image registration is employed to propagate structures from a reference image onto the session MR images. Then, selective re-contouring is performed on specific areas, primarily within the planning target volume plus a 2 cm margin (PTV+2 cm), to adapt to anatomical changes. This approach significantly reduces the manual re-contouring time to 10–20 min. Developing robust auto-segmentation methods to speed up this contouring process is needed to improve efficiency in the MR-Linac workflow. Various artificial intelligence (AI) methods with different architectures and techniques have been introduced to improve segmentation performance (Çiçek *et al* 2016, He *et al* 2016, Badrinarayanan *et al* 2017, Chen *et al* 2018, Isensee *et al* 2018, Myronenko 2019, Hsu *et al* 2021). However, determining the best-performing auto-segmentation model for images acquired from MR-Linac machines, particularly concerning pancreatic cancer patients, remains an area of ongoing investigation.

Although auto-segmentation algorithms have seen significant advancements, unresolved challenges remain due to complexities from tumor and organ size, shape, and position variations, contributing to segmentation inaccuracies that impact RT precision (Ghorpade *et al* 2023). Additionally, the diverse range of imaging modalities and contrasts necessitates the development of specific models tailored to each. In the treatment of pancreatic cancer patients using MR-Linacs, abdominal motion presents significant challenges. To address this, the acquisition of 3DVane images (utilizing stack-of-stars acquisition (Zhou *et al* 2017)—Philips Co.) has become current standard practice on Unity MR-Linacs. 3DVane images are motion-averaged representations derived from 4D MR images and therefore present a trade-off between image contrast and motion artifacts (Chandarana *et al* 2011). This inherent characteristic makes the manual contouring process on 3DVane images compared to other MR imaging techniques more difficult and labor-intensive. Auto-contouring remains difficult even with breath-hold techniques, due to several factors such as significant day-to-day anatomical variations producing unpredictability in organ positioning and shape.

The development of deep learning-based auto-segmentation algorithms for 3DVane images and even images with breath-hold techniques is severely constrained by the scarcity of available data. Data augmentation has shown to be critical in mitigating this issue, enabling optimal utilization of available datasets (Fabian *et al* 2021). These techniques encompass a spectrum of approaches, including spatial and intensity transformation (Hinton *et al* 2012, Krizhevsky *et al* 2012, AbuSalim *et al* 2022, Anaya-Isaza and Mera-Jiménez 2022, Bar-David *et al* 2023), or using generative adversarial networks (GANs) for synthetic image generation (Frid-Adar *et al* 2018, Huang *et al* 2018, Hammami *et al* 2020) to increase data count. Nonetheless, conventional data augmentation methods, like rotation and flipping, show limited impact as they are usually applied globally or unrealistically. Therefore these methods must be sufficiently complex and sophisticated to mimic more realistic data.

While transformation techniques offer one approach to expanding the training data size, borrowing data from a different modality could be another strategy. GANs have shown potential in cross-modal data borrowing by synthesizing images that closely resemble the target modality. However, the application of GANs for synthetic MRI generation faces several challenges such as mode collapse where the generator produces limited variations, the introduction of unrealistic artifacts, and a strong dependence on both the quantity and quality of the training dataset (Goodfellow *et al* 2020).

So far all these attempts have their particular pitfalls which make them unsuitable for our problem at hand. Additionally, there is currently no commercially available auto-contouring algorithm specifically designed for 3DVane images acquired on MR-Linacs that addresses this problem. This gap underscores the critical need for developing an auto-contouring model.

We, therefore, propose a novel structure-guided deformation-based approach (sgDefAugs) that goes beyond conventional augmentations (ConvAug) by generating realistic images through targeted spatial transformations, while remaining computationally efficient and sufficiently flexible for representing local deformations. Additionally, we aim to present a cross-modality approach by generating synthetic 3DVane images from CT scans using a previously implemented PatchGAN discriminator CycleGAN (Isola *et al* 2017, Shojaei *et al* 2024) and perform fine-tuning on the weights by re-training on the real data to reduce the impact of unrealistic artifacts. Alongside these augmentation approaches, our goal is to identify the best-performing segmentation model for OAR contouring in pancreatic cancer. We focus on evaluating previously-implemented top-performing models-namely nnU-Net, SegResNet, and ResU-Net-trained on 3DVane images, to determine which model performs best in scenarios with limited training data availability. Subsequently, our other aim is to present a pipeline that integrates the best-performing segmentation and augmentation approaches to produce auto-contours that can potentially streamline the current online adaptive workflow.

## 2. Materials and methods

This study comprises two sequential phases. In phase I, we customized 3 top-performing models from the literature for our application and then determined the best-performing model. Subsequently, in phase II, advanced augmentation techniques were implemented to investigate if the performance of the best-performing model could be further improved. This process was tailored to achieve high-quality results even with a small dataset, addressing the common challenge of limited data availability in medical imaging research.

### 2.1. Data selection

The dataset for training the auto-segmentation model and the GAN approach contained various MR and CT imaging data and contours of OARs from pancreatic cancer patients who were treated on a 1.5 T Unity MR-Linac. These were requested from the MOMENTUM study (de Mol van Otterloo *et al* 2020). All imaging data in this study used the DICOM coordinate system. For each patient, up to four 3DVane image sequences were potentially available: T1-weighted and balanced sequences, each with and without spectral attenuated inversion recovery. At our institute, for imaging pancreatic cancer patients and proceeding with treatment, clinicians select a single sequence on a patient-specific basis, prioritizing optimal structure visibility. Due to the availability of more images with balanced contrast, we chose to base this study on balanced 3DVane (b3DVane) images. The imaging protocol incorporates the use of a compression belt which restricts breathing motion. After consultation with clinicians and physicists at our institutes and reference to prior research (Chen *et al* 2020, Lukovic *et al* 2020), the dataset was consolidated into 8 OARs essential for online adaptive RT, which include the duodenum, liver, left kidney, right kidney, spleen, stomach, spinal canal, and small bowel. Hence, pre-treatment CT and on-treatment b3DVane MR images with structures from 10 patients over 43 sessions were obtained to train the auto-segmentation and GAN models.

Another dataset, a publicly available pediatric CT dataset with expert contours for 29 structures and 325 images, was utilized to synthetically generate b3DVane images via generative networks (Jordan *et al* (2022)). This dataset was selected because it was the only available dataset containing all 8 OARs of interest.

Our models were trained on the OAR ground-truth contours derived from modified contours obtained during the online adaptive RT workflow. However, for the evaluation of our models’ predictions, the OARs in the reported images were manually contoured and independently validated by two clinicians.

### 2.2. Image preprocessing

After converting the DICOM files to images and binary labels using Plastimatch (Sharp *et al* 2010), we cropped them to include only the foreground regions, i.e. those that contained organs of interest. The background, which included arms and support structures, was automatically excluded (Ibanez *et al* 2003). Some of the structures were overlapping each other. To resolve this, overlaps were automatically removed by assigning each voxel within the overlapping area to the closest of the non-overlapping structures. To address intensity inhomogeneities, artifacts, and noise in MR images, N4 bias field correction (Tustison *et al* 2010) and anisotropic diffusion filtering (Perona and Malik 1990) were applied respectively using SimpleITK (Ver. 2.1.1.2) (Sing *et al* 2015). The parameters for these tasks were taken from Arabi and Zaidi (2016). With regards to the computational memory capacity, the original images were resized from 640 × 640 × 147 voxels to 448 × 448 × 128 voxels for ResU-Net and SegResNet. Subsequently, intensity normalization was performed by re-scaling the image values to the -1 to 1 range to ensure consistent and faster learning.

### 2.3. Phase I—model selection

Three 3D models were trained and evaluated: ResU-Net, SegResNet (Chen *et al* 2018, Myronenko 2019, Hsu *et al* 2021), and nnU-Net (Isensee *et al* 2018).

#### 2.3.1. Conventional on-the-fly data augmentation approach

During training, a comprehensive data augmentation approach, referred to as ConvAug methods in this study, was implemented with the following steps. For the SegResNet and ResU-Net models, three patches, each with dimensions of 240 × 240 × 80 voxels, were randomly extracted from the 3D images and structures. Random Rician and Gaussian noise, along with random contrast adjustment and intensity shifts, were applied to the training patches. To improve robustness, elastic deformations—non-linear spatial transformations randomly applied to an image, without anatomical constraints—were used. All augmentations in this step were executed using the Monai Python library, version 1.3.0 (Cardoso *et al* 2022).

The same ConvAug were applied to the nnU-Net training dataset, with a patch size of 192 × 192 × 64. Since nnU-Net is a self-configuring framework, spatial information was determined automatically by the model.

#### 2.3.2. Model parameters

**Figure A1.**
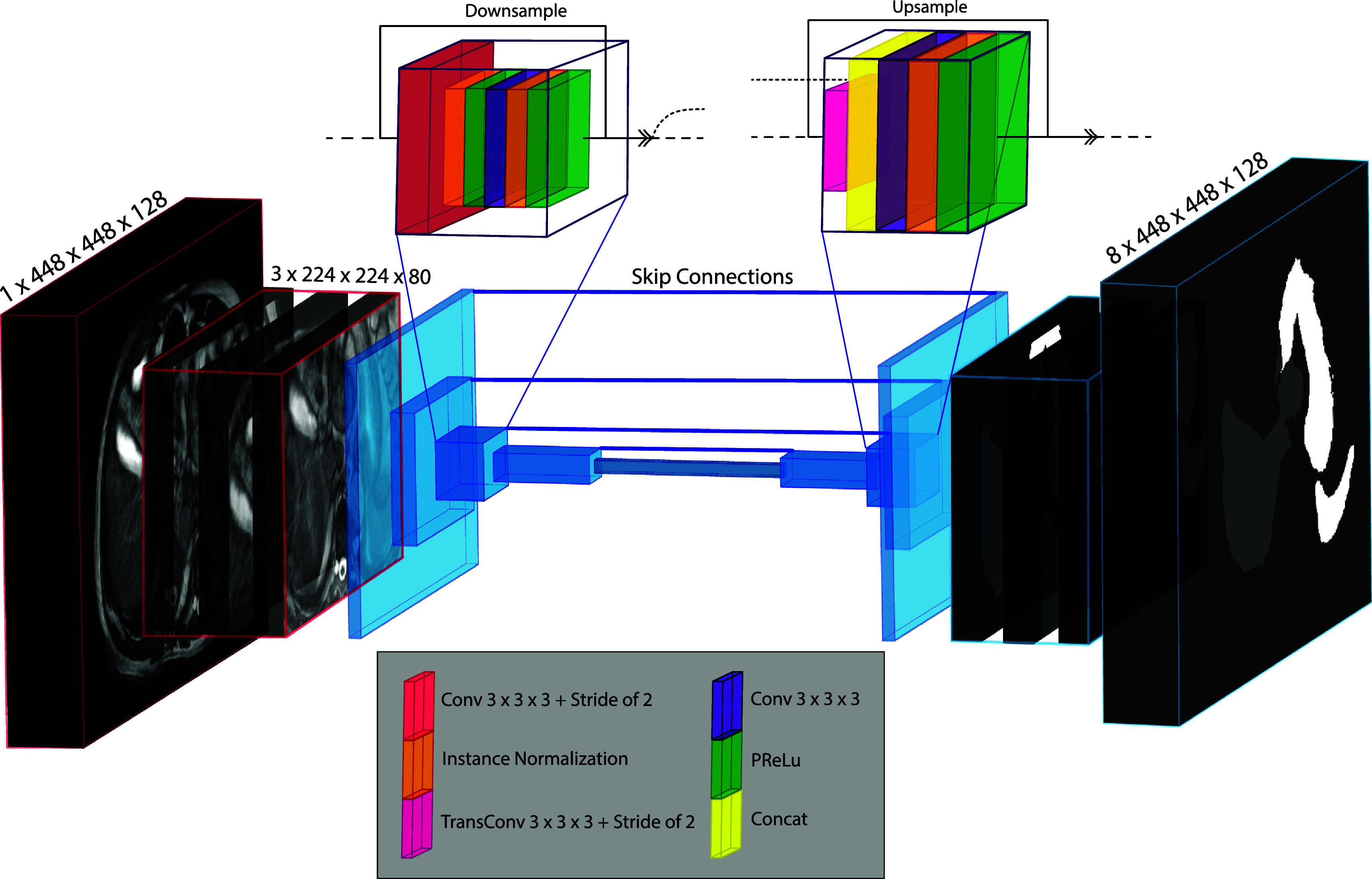
The customized architecture of the 3D ResU-Net implemented in our study. The network consists of encoder and decoder paths, with each block (depicted in blue) comprising 3D convolutions, instance normalization, and PReLu activation function. The color-coded components in the top panel detail the layers within each block. The model takes three input patches and generates an 8-channel label output.

**Figure A2.**
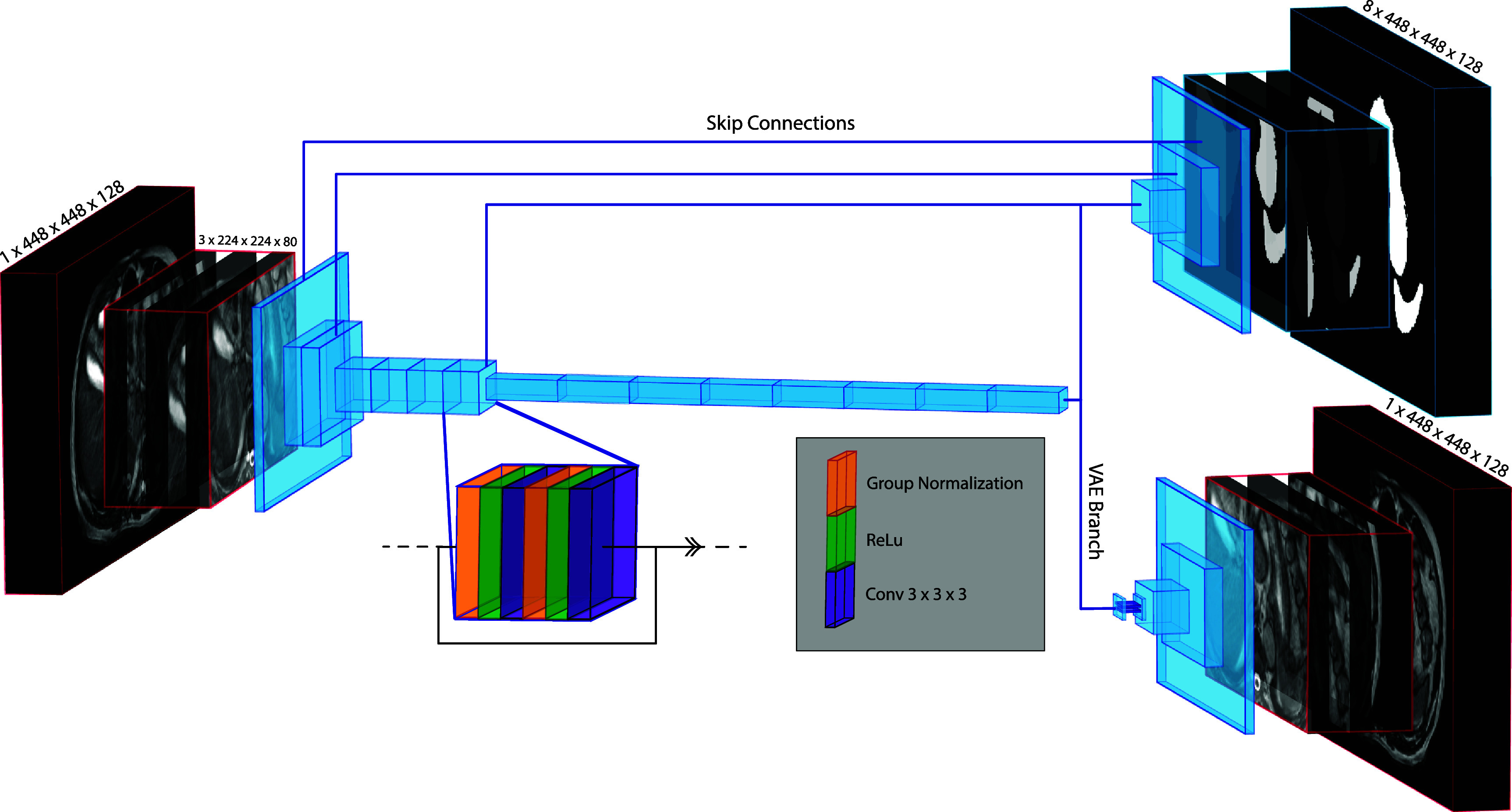
The customized architecture of the 3D SegResNet implemented in our study. The network consists of an encoder and two decoder paths, with each block (depicted in blue) comprising 3D convolutions, group normalization, and ReLu activation function. The color-coded components in the bottom panel detail the layers within each block. The model takes three input patches and generates an 8-channel label output. The VAE branch (on the bottom) is only used during the training process to regularize the segmentation output by reconstructing itself and incorporating a penalty into the segmentation loss function.

The architectures of our customized ResU-Net and SegResNet models are illustrated in figures A1 and A2. Further details regarding the model configurations are provided in section A.1.

A weighted average of Dice loss (Ma *et al* 2021) and cross-entropy loss (Goodfellow *et al* 2016) was used as the objective function; more information regarding the selection of the loss function is provided in section A.1. Model optimization was performed using the Adam algorithm, with a learning rate of 1 × 10^-4^ and weight decay of 1 × 10^-5^ to regularize and prevent over-fitting. To dynamically adjust the learning rate during training, the cosine annealing learning rate scheduler was employed (Loshchilov and Hutter 2016). The models were trained on a 24 GB NVIDIA RTX A5000 GPU for 200 epochs. A five-fold cross-validation was implemented at the patient level rather than the image level to prevent different folds containing similar images from the same patient.

### 2.4. Phase II—dataset augmentation

After identifying the best-performing auto-segmentation model based on geometric accuracy (as explained in section 2.5.1), we implemented two dataset augmentation approaches to investigate if the model’s performance could be further improved: GANAugs-based augmentations and local non-rigid sgDefAugs. These methods were applied in addition to the baseline ConvAug approach, previously described in section 2.3.1. Our objective was to assess whether expanding the dataset using GANAug and sgDefAug, while retaining ConvAug, could further enhance the model’s performance. To evaluate the proposed augmentation approaches, we selected the best-performing fold from the initial model selection explained in 2.3. Our aim was to determine if we could enhance the model’s performance using the same fold.

#### 2.4.1. sgDefAug approach

The primary objective of this approach was to induce controlled and local spatial distortions within each labeled OAR, simulating anatomical variability across different patients. To implement this task, we employed Platipy, a tool that facilitates localized image deformation based on organ-specific transformations. Platipy allows users to apply targeted deformations to regions of interest within an image (Chlap and Finnegan 2023). The deformation process was applied to each anatomical structure within each MR/CT image sequentially, effectively impacting the entire organ’s shape and consequently changing the image. The sgDef process started with the selection of an initial structure. This structure underwent two transformations: a random displacement, which shifted it from its original position, and a random scaling factor, which either shrank or expanded its size. The resulting structure-specific transformation was applied to the image, deforming the selected structure and the other surrounding structures. Subsequently, a second structure was selected from the transformed structures, and the process was repeated. This procedure continued iteratively until all structures were processed. The final transformation was obtained by composing all transformations from each sequential step. This process enabled the application of sgDef, ensuring that each structure’s unique deformation was accounted for in the overall deformation of the image.

The extent of deformations was controlled by random variables from uniform distribution within a predefined interval along the *x, y,* and *z* coordinates, ensuring that the deformations remained anatomically plausible. The interval thresholds were set to 40 mm for both the displacement and shrinkage/expansion variables. The rationale behind these threshold values was to ensure that the deformations applied to the medical image data remain within reasonable limits, avoiding excessive distortions. The choice of these thresholds was confirmed through visual inspection of the resulting deformed images to ensure avoidance of implausible shape distortions. A Gaussian smoothing filter with a standard deviation of 5 mm was applied to the deformation vector fields of the transformed structures. This filtering process reduced any abrupt or jagged contours that could potentially emerge during the deformation process. By applying these random spatial deformations, we expanded our dataset from the initial 43 images from 10 patients to a total of 430 images. This augmented dataset was then used to train the best-performing auto-segmentation model for 400 epochs.

#### 2.4.2. GANAug approach

This approach was implemented to synthesize plausible b3DVane images from CT scans with ground-truth contours to train our best-performing model with more data. To develop this model, we used all 43 b3DVane images with their corresponding CT scans. Each patient case included a single CT scan and multiple session b3DVane images.

To facilitate training convergence for generating plausible medical images, each patient’s CT scan was registered onto their corresponding session MR images. The registration was performed in two sequential steps. First, an affine registration was employed to globally align anatomical structures. Subsequently, a B-spline deformable image registration was added to account for residual local deformations to align the images as much as possible. The image registration was implemented through Elastix, (Klein *et al* 2009), and SimpleITK (Python). We expanded our dataset using the sgDefAug approach, as detailed in section 2.4.1. These processes resulted in a total of 244 pairs of CT and MR images, which were input into the generative model.

**Figure A3.**
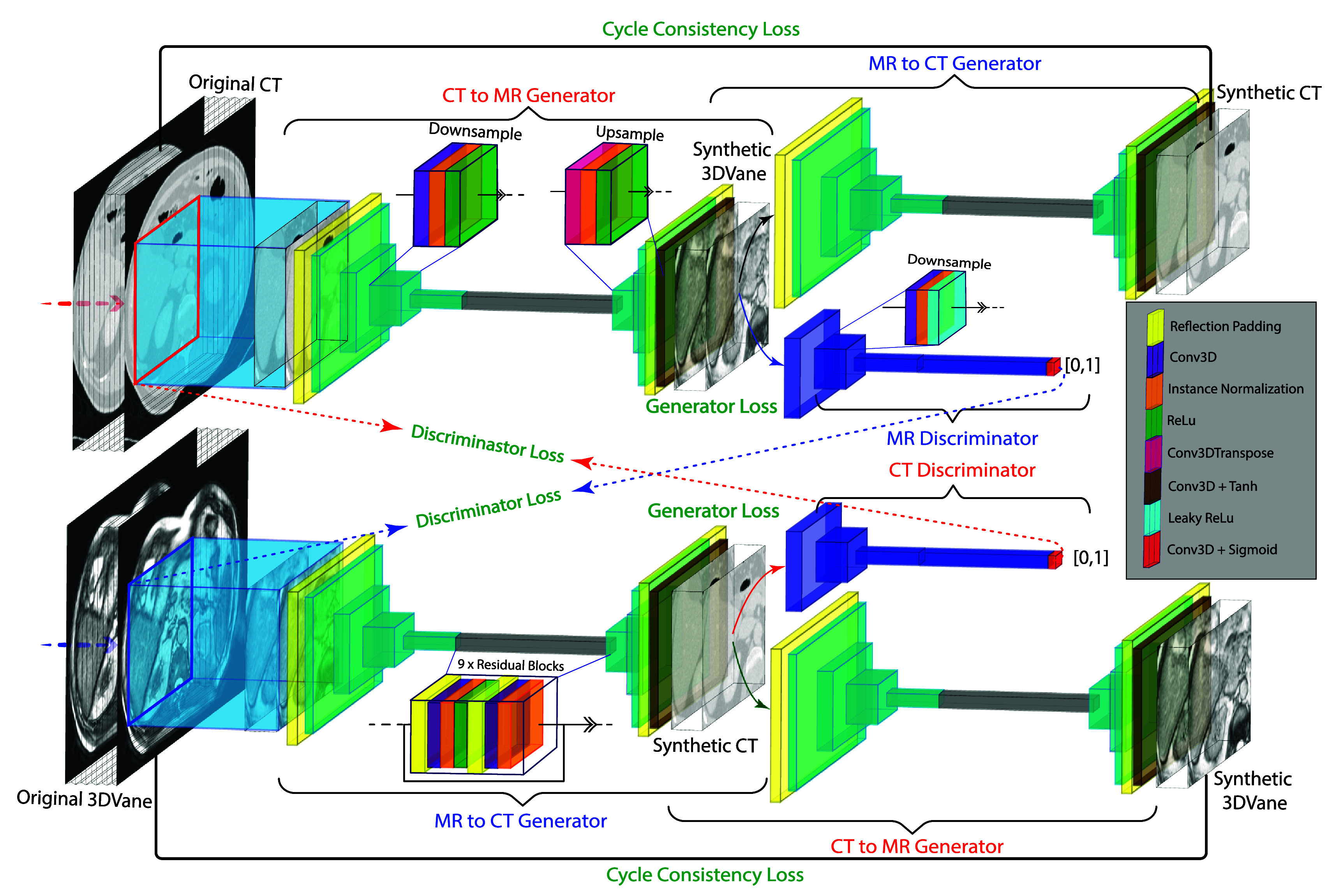
The architecture of the implemented cycleGAN, highlighting the different loss functions used. The top architecture represents the cycle for CT$\rightarrow$MR$\rightarrow$CT, while the bottom architecture represents the cycle for MR$\rightarrow$ CT$\rightarrow$MR. Each block (depicted in green) comprises specific components for the generators and discriminator. For the generators, each block comprises 3D convolutions, instance normalization, and ReLU activation, with a tanh activation function in the final layer. The discriminator blocks similarly include 3D convolutions and instance normalization, but have leaky ReLU activation, with a sigmoid activation function in the final layer.

A cycleGAN model featuring a patchGAN discriminator was implemented. The cycleGAN architecture, with its cycle consistency loss, is the preferred approach for image-to-image translation, ensuring reversible transformations and realistic images across modalities like CT-to/from-MR. Additionally, the patchGAN component of the implemented architecture processes local image patches, preserving texture and structure by penalizing inconsistencies at the patch level, requiring fewer parameters, and accelerating training. These factors make PatchGAN-based CycleGAN both efficient and effective for medical imaging applications (Isola *et al* 2017, Isola_2017_CVPR, zhu2017unpaired„ wang2023dc, daml24), Ni 2024. Figure A3 represents the architecture design and elements used to implement this network. Further details regarding the model configuration are provided in section A.1. The generator and discriminator loss functions were calculated using mean squared error (MSE) (Welander *et al* 2018). The cycle consistency loss, which measures the error between the reconstructed and the original images, was computed using a weighted average of one minus the structural similarity index measure (SSIM) and the the mean absolute error (MAE) (Wang *et al* 2004, Willmott and Matsuura 2005). SSIM enforces image similarity in the cycle consistency loss to preserve fidelity between CT and MR domains, addressing potential artifact problems and mode collapse issues that can arise in GAN models (Pai *et al* 2023). The identity loss, which measures how well each generator preserves the input image when applied to images from its target domain, was also calculated using MAE. Adam algorithm, with a learning rate and weight decay of 1 × 10^–4^ and 1 × 10^–5^ was used, respectively, to avoid overfitting. The cosine annealing learning rate scheduler was employed to adjust the learning rate (Loshchilov and Hutter 2016).

The model was trained on a 24 GB NVIDIA RTX A5000 GPU for 1000 epochs. We monitored the outputs visually throughout the training to determine the optimal point to stop the process to avoid over/underfitting. The dataset was randomly split into training and validation sets with a ratio of 80% for training and 20% for validation. To further mitigate artifacts and mode collapse, we conducted thorough visual inspections to detect irregularities. The model architecture design and hyperparameters were also carefully selected, and the training process was repeated multiple times.

In the inference step, the pediatric abdominal CT images detailed in 2.4.1 were employed to generate synthetic b3DVane images. As a result, 325 images were synthesized. The generated images were used to train our best-performing auto-segmentation model for 1000 epochs. Any potentially unseen remaining artifact issues were minimized by updating the auto-segmentation model through a fine-tuning process. This crucial step aimed to refine the model based on real images and mitigate any ambiguities introduced by the cycleGAN in the synthetic b3DVane images. Therefore, the auto-segmentation model was trained on real b3DVane images from the training data of the 5-fold cross-validation for another 10 epochs.

### 2.5. Evaluation experiments

To validate our auto-contours, we implemented a comprehensive analysis incorporating both geometric and dosimetric criteria. While geometric analyses provide valuable insights, they may not always be the best indicators of clinical utility in RT. Therefore, we supplemented our evaluation with dosimetric analysis, which offers a more direct measure of potential clinical impact.

#### 2.5.1. Geometric analysis

The Dice score was calculated to quantify the overlap between predicted and ground truth segmentations (Zou *et al* 2004), while the average surface distance (ASD) was used to measure the mean distance between the surfaces of the predicted and ground truth contours (Yeghiazaryan and Voiculescu 2018). These complementary metrics provide a comprehensive assessment of both volumetric similarity and boundary accuracy.

#### 2.5.2. Dosimetric analysis

We evaluated a previously generated treatment plan based on the ground-truth contours of a random test image from the best fold and obtained the dose distribution. Then, we derived dose-volume histogram (DVH) curves for all organs, employing both the ground-truth and predicted contours. Subsequently, we conducted a comparative analysis of the DVH criteria, specifically the D2% and D50% metrics from the predicted and ground truth contours. These metrics were selected because D2% represents the near-maximum dose received by an organ, while D50% indicates the dose received by 50% of the volume, i.e. providing the median dose distribution.

The treatment planning was performed using Elekta’s (Stockholm, Sweden) Monaco^®^ treatment planning system version 5.4, with 5 fractions and a total dose of 40 Gy. The patient had been previously treated on the Unity MR-Linac using the intensity-modulated RT (IMRT) technique.

## 3. Results

The subsequent sections present a comparative analysis of the results, first for the various investigated auto-segmentation models, and then for the augmentation approaches, based on geometric and dosimetric criteria, supported by statistical analysis.

### 3.1. Model selection

The training duration for ResU-Net and SegResNet was approximately 30 h, whereas nnU-Net reduced the training time substantially, requiring only 5 h. For inference, the model generates predictions in approximately 30 s when bias field correction and denoising filters are applied. The majority of the inference time (25 s) was taken up by these two steps.

Table 1 presents a comparative analysis of the performance metrics for various trained models. The nnU-Net (mean Dice score: 0.78 ± 0.10, mean ASD: 3.92 ± 1.94 mm) demonstrated superior performance compared to other models based on these criteria. The SegResNet (mean Dice score: 0.72 ± 0.14, mean ASD: 5.90 ± 2.29 mm) yielded marginally more accurate predictions than the ResU-Net (mean Dice score: 0.68 ± 0.19, mean ASD: 8.26 ± 6.08 mm). Regarding the organ-specific segmentation accuracy, almost a consistent pattern was observed across all models. The liver had the highest Dice score, while the small bowel demonstrated the lowest. Analysis of the ASD showed that the liver had the least error in both the SegResNet and nnU-Net models. However, the ResU-Net model showed the lowest error for the right kidney. The highest ASD was consistently obtained in the small bowel across all models.

**Table 1.** Comparison between the outputs of the different auto-segmentation models and the ground-truth contours. nnU-Net consistently outperforms other models across the majority of the evaluated structures. Better performance (higher Dice and lower ASD) is indicated in bold.

	Dice			ASD (mm)		
Organs	ResU-Net	SegResNet	nnU-Net	ResU-Net	SegResNet	nnU-Net
Liver	0.88	0.87	**0.96**	3.94	2.73	**1.36**
Duodenum	0.40	0.49	**0.65**	17.34	6.51	**3.25**
L. Kidney	0.83	**0.85**	0.78	5.28	**5.01**	5.61
R. Kidney	0.82	0.80	**0.83**	3.12	3.25	**1.70**
Spinal Canal	0.75	0.78	**0.82**	3.42	4.52	**2.96**
Spleen	0.75	0.75	**0.76**	7.25	8.94	**6.15**
Small Bowel	0.35	0.52	**0.63**	19.65	9.28	**6.93**
Stomach	0.68	0.71	**0.77**	6.08	6.94	**3.37**

Mean	0.68	0.72	**0.78**	8.26	5.90	**3.92**

**Figure 1.**
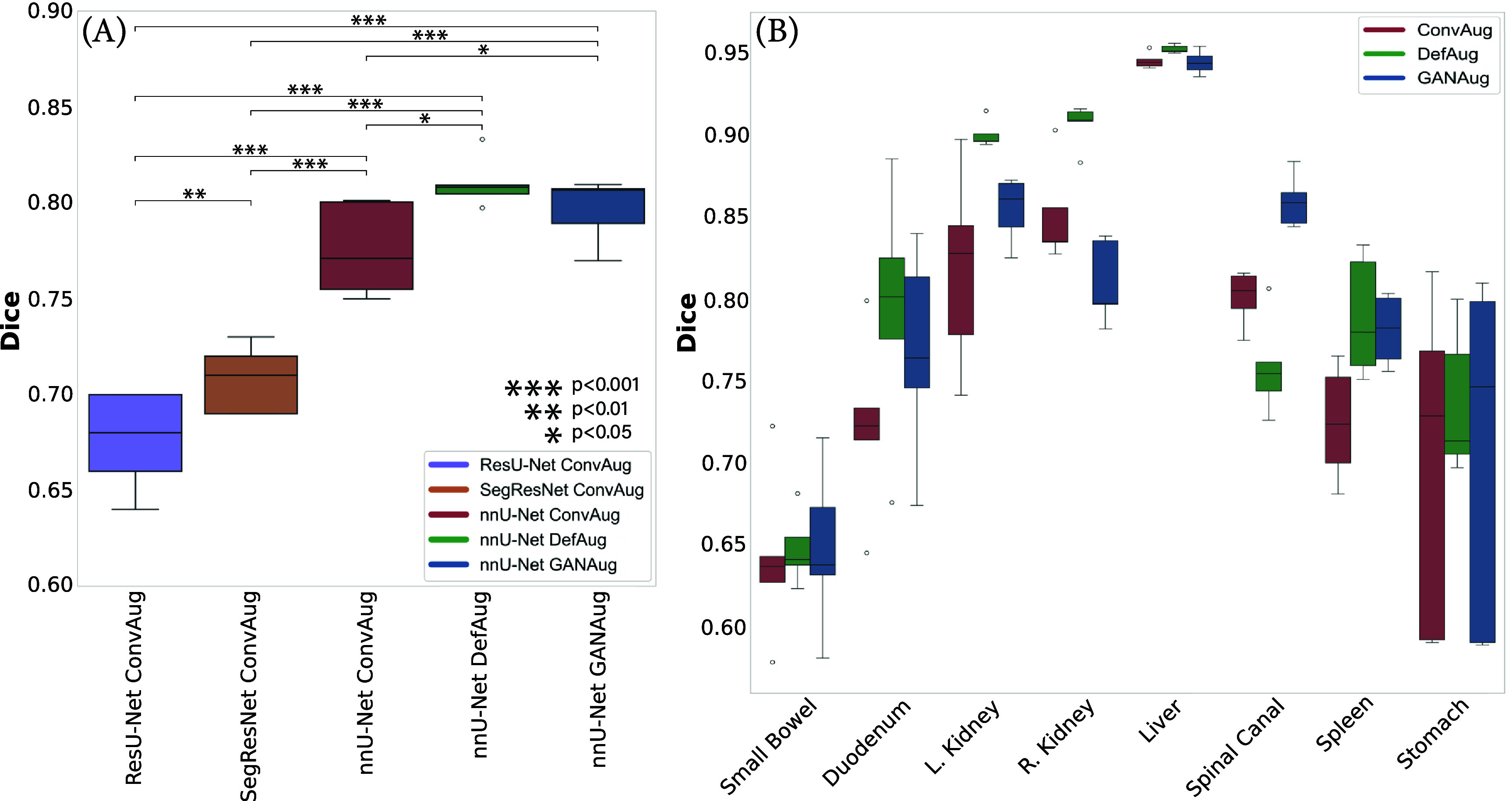
Comparison of augmentation approaches and auto-segmentation models: (A) across patients and (B) across anatomical structures. Statistical significance levels are denoted by asterisks. The nnU-Net framework demonstrates statistically significant superior performance compared to ResU-Net and SegResNet. The sgDefAug and GANAug approaches indicate improved Dice scores for all structures compared to conventional methods.

A student’s t-test was conducted to assess the statistical significance of differences between segmentation approaches in terms of Dice scores. Figure 1(A) (the first three columns on the left for phase I results) illustrates the comparative performance of these appraoches across the test image set. The analysis showed statistically significant differences among all auto-segmentation models. Specifically, the nnU-Net demonstrated significantly superior accuracy compared to other auto-segmentation frameworks (*p* < 0.001). Additionally, the SegResNet had significantly better performance than the ResU-Net (*p* < 0.01).

### 3.2. Augmentation approaches

Given that nnU-Net demonstrated superior performance among the considered frameworks, subsequent investigations focused on improving its performance with the available limited dataset by leveraging advanced augmentation techniques. ConvAug with nnU-Net was established as the baseline for comparison with our proposed approaches.

The training process of the cycleGAN required 5 d. Following this, training the best-performing auto-segmentation model using synthetic b3DVane images generated by the cycleGAN took 20 h. However, the fine-tuning step was performed in only 2 h. In contrast, training the nnU-Net model with the generated images from the sgDefAug approach took approximately 8 h. This is significantly faster than the training time for the GANAug approach, which required a factor of 18 more time to complete.

**Figure 2.**
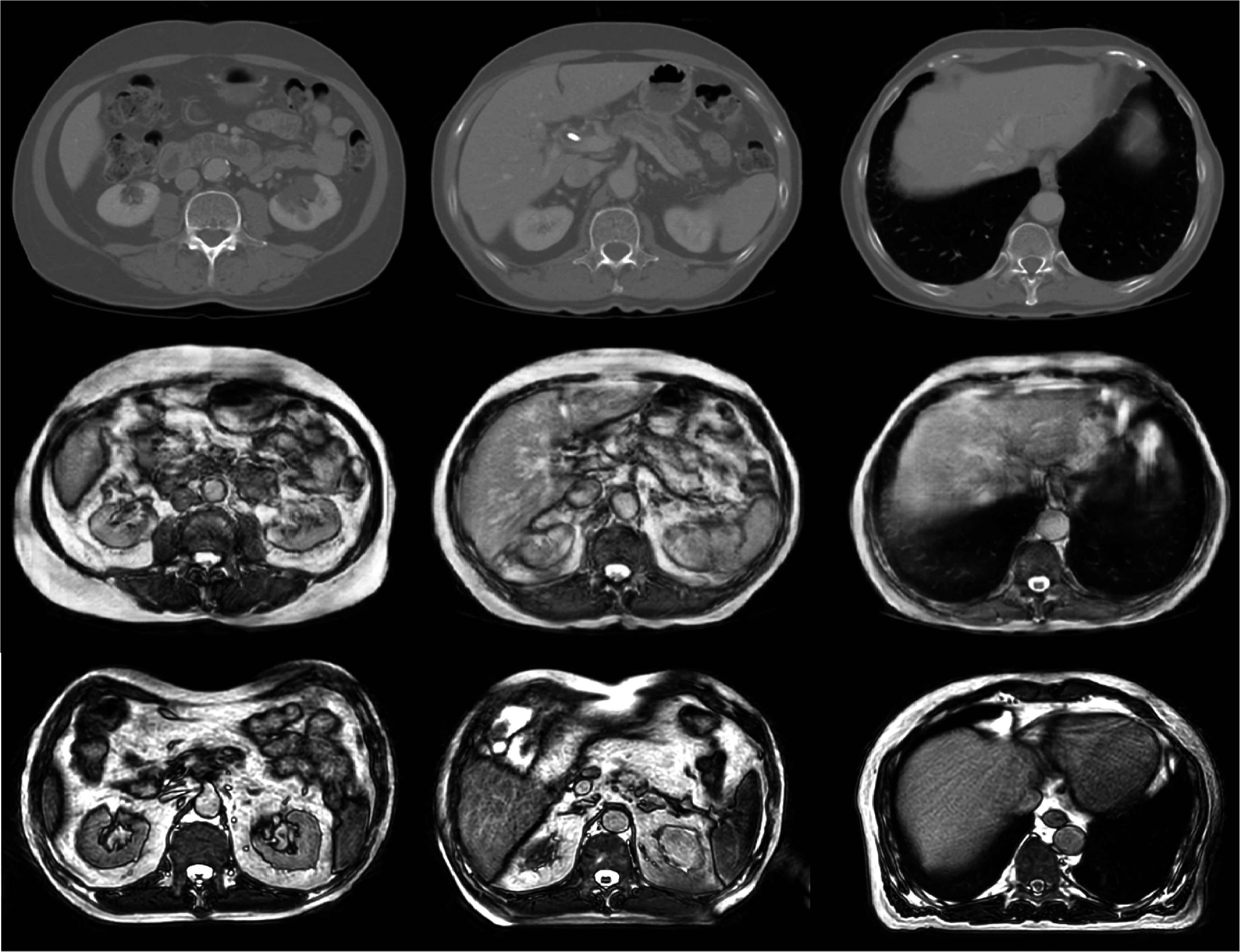
Abdomen CT scans (top), generated b3DVane images (middle), and real examples of b3DVane images (bottom). This figure illustrates the similarity in soft-tissue contrast between the generated and real images.

**Figure 3.**
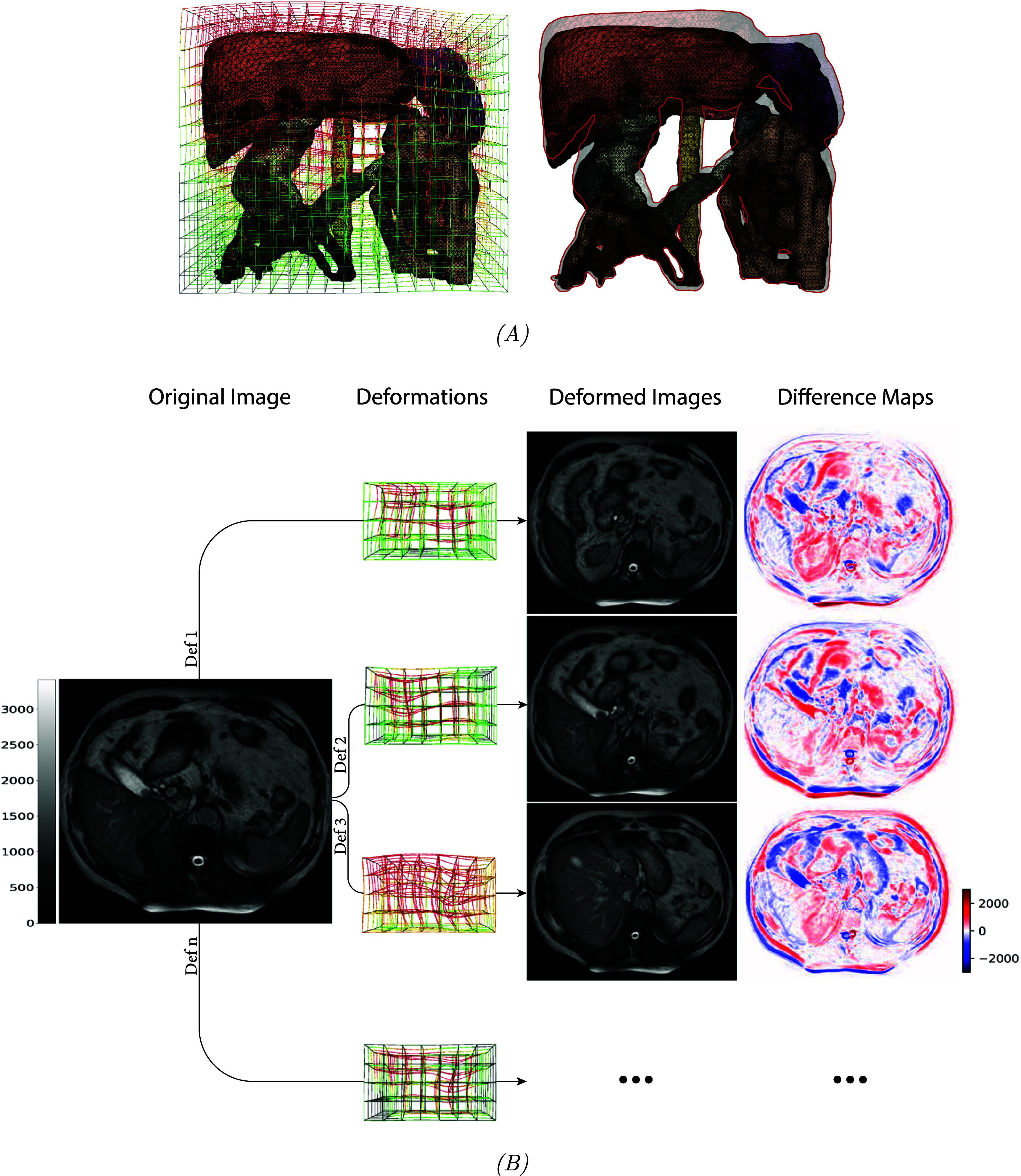
A 3D-rendered mesh of anatomical structures before deformation (A—left), overlaid with a random deformation vector field generated from multiple sequential deformations/displacements, alongside its resultant deformed output (A—right). The deformed structures are visualized with reduced opacity and contoured in red. (B) Presents the same slice from the same patient, demonstrating the impact of these deformations on the image while avoiding unrealistic distortions. In this example, there are substantial changes in the shapes of the liver and stomach after deformation, while the spleen remains relatively unaffected due to the random nature of the deformations. Difference maps highlight significant variations along the x, y, and z axes, with color bars indicating the range of MR image intensities and the degree of differences represented in the maps.

The resultant image outputs from each advanced augmentation approach are illustrated in figures 2 and 3. Specifically, figure 2 demonstrates output examples from the trained cycleGAN model, which generated 325 synthetic b3DVane images with contrast characteristics similar to real images. Whereas, figure 3 presents examples of deformed images from the sgDefAug approach, along with their corresponding difference maps. These maps reveal substantial variations in organ location and size on the same slice of the same patient post-deformation, indicating effective data augmentation. A clinician verified the realism and anatomical plausibility of the generated images.

**Figure 4.**
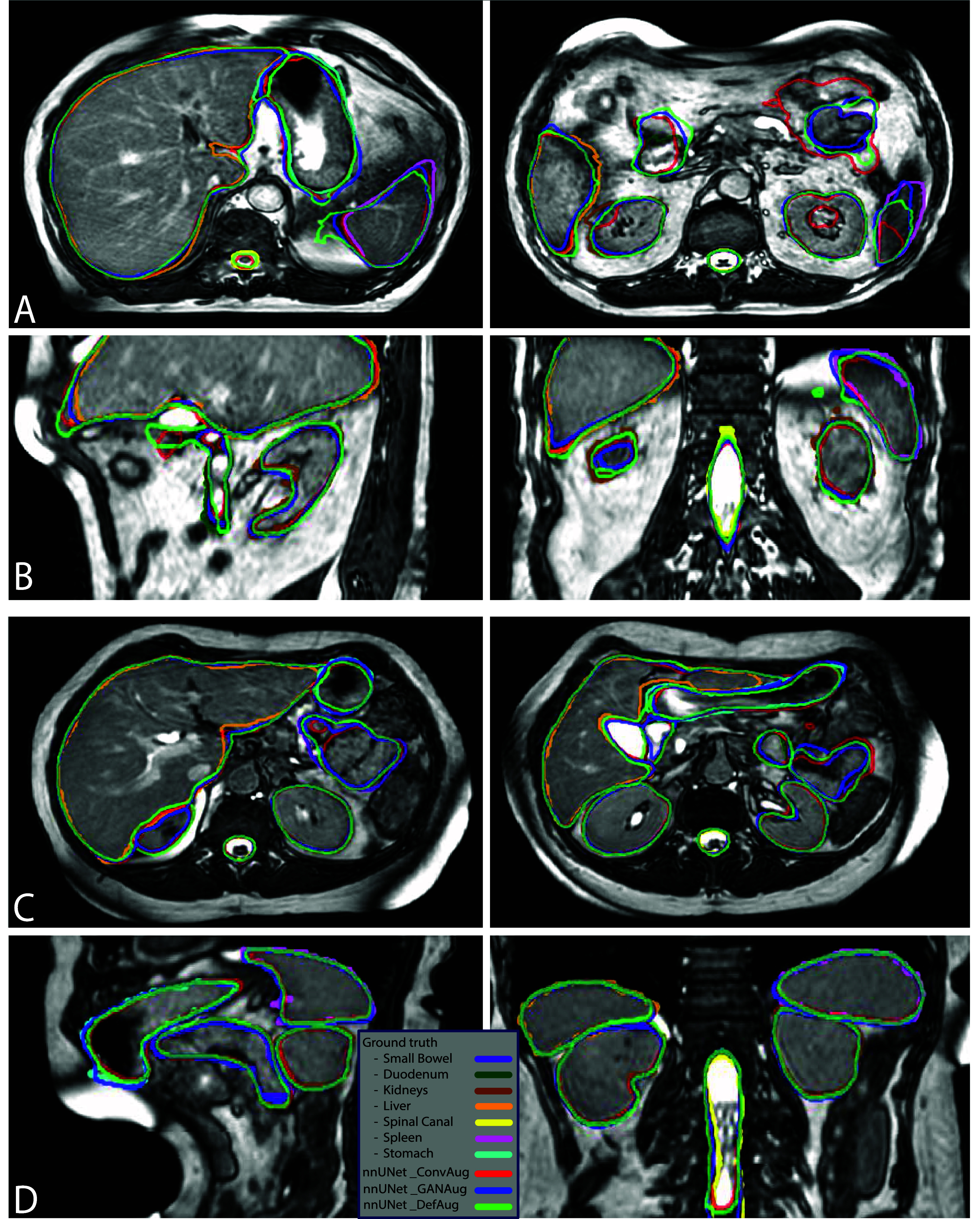
A comparison of outputs from various augmentation approaches against the ground truth on two patients in different imaging planes. (A) and (B) Demonstrate a scenario in which the ConvAug approach underperforms in accurately predicting the small bowel, while the other approaches show results that are closer to the ground truth. (C) and (D) Illustrate a good agreement between the predictions and the ground truth contours along all OARs.

Figure 4 depicts the auto-contour outputs of various approaches for two patients from the validation dataset. The GANAug and sgDefAug approaches demonstrate good alignment with the ground truth, whereas the ConvAug approach has notable inaccuracies in certain parts. Specifically, figure 4. A shows a case where the model trained with the ConvAug approach produced noticeably inaccurate contours for the small bowel, duodenum, and spleen.

Figure 1(A) and table 2 present a comparative analysis of Dice scores and ASD across different approaches. Statistical analysis reveals significant differences between GANAug (mean Dice score: 0.82 ± 0.09, mean ASD: 3.38 ± 1.82 mm) and sgDefAug (mean Dice score: 0.84 ± 0.09, mean ASD: 3.14 ± 1.79 mm) approaches when compared to ConvAug (mean Dice score: 0.78 ± 0.10, mean ASD: 3.92 ± 1.94 mm), both with *p*-values $ < $ 0.05. No statistically significant difference was observed between GANAug and sgDefAug approaches. Consistently across all approaches, the liver and small bowel had the highest and lowest Dice scores, respectively. An inverse pattern was observed in the ASD evaluation. Figure 1.B depicts the comparative performance of different augmentation approaches and the resulting improvement for each organ. The sgDefAug and GANAug approaches improved the performance of the nnU-Net for the majority of organs. The sgDefAug approach resulted in a smaller standard deviation of the results, indicating more consistent high-performance predictions.

**Table 2.** Comparison of the outputs of the data augmentation approaches employed with nnU-Net and the ground truth contours. Both sgDefAug and GANAug approaches consistently demonstrate superior performance compared to the ConvAug approach across all evaluated structures, with the sgDefAug approach showing slightly better performance than the GANAug approach. Better performance (higher Dice and lower ASD) is indicated in bold.

	Dice			ASD [mm]		
Organs	ConvAug	GANAug	sgDefAug	ConvAug	GANAug	sgDefAug
Liver	**0.96**	**0.96**	**0.96**	1.36	1.37	**1.34**
Duodenum	0.65	0.68	**0.80**	3.25	2.82	**2.60**
L. Kidney	0.78	0.88	**0.92**	5.61	**1.4**	1.50
R. Kidney	0.83	0.84	**0.91**	**1.70**	2.56	1.64
Spinal Canal	0.82	**0.86**	0.80	2.96	**2.79**	3.16
Spleen	0.76	0.81	**0.83**	6.15	4.41	**4.10**
Small Bowel	0.63	**0.68**	0.65	**6.93**	7.26	7.15
Stomach	0.77	**0.81**	0.80	**3.37**	4.41	3.65

Mean	0.78	0.82	**0.84**	3.92	3.38	**3.14**

The differences in D2% and D50% dosimetric criteria across the predictions from the implemented augmentation approaches, based on the DVH curves for all structures, are presented in table 3. This table provides a comparison of how each approach’s predictions align with the ground truth in terms of high-dose and median-dose distributions across the anatomical structures. The values of the dosimetric criteria calculated on the contours generated by the sgDefAug approach were more similar to the ground truth values derived from the manual structures than for other approaches. Specifically, 75% of the organs had D2% and D50% differences of less than 1% with sgDefAug approach. On the other hand, both the ConvAug and GANAug approaches resulted in 62.5% and 37.5% of organs showing D2% and D50% differences of less than 1%, respectively. Notably, no organs in the sgDefAug approach demonstrated D2% differences greater than 5%. In contrast, 37.5% of organs in the ConvAug approach and 25% in the GANAug approach showed D2% differences greater than 5%. The ConvAug approach shows substantial discrepancies compared to the ground-truth D2% values for the stomach, duodenum, and small bowel.

**Table 3.** Comparison of D2% and D50% differences between outputs from various approaches, stratified into three categories for simpler interpretability. The reported values are expressed as percentages. The sgDefAug approach demonstrates results with greater dosimetric similarity to the ground truth.

Dose Difference Percentage: [ΔD<1%: ✔], [1%<ΔD<5%: •], and [5%<ΔD: ✘ ]						
	D2% Difference			D50% Difference		
Organs	ConvAug	GANAug	sgDefAug	ConvAug	GANAug	sgDefAug
Liver	0.30 ✔	0.89 ✔	0.61 ✔	0.01 ✔	0.03 ✔	0.01 ✔
Duodenum	13.85 ✘	9.20 ✘	2.67 •	9.64 ✘	10.10 ✘	8.51 ✘
L. Kidney	0.03 ✔	0.59 ✔	0.07 ✔	2.20 •	1.22 •	0.49 ✔
R. Kidney	0.28 ✔	0.13 ✔	0.09 ✔	1.81 •	3.73 •	0.11 ✔
Spinal Canal	0.19 ✔	0.44 ✔	0.40 ✔	1.01 •	1.07 •	2.90 •
Spleen	0.97 ✔	0.99 ✔	0.44 ✔	0.05 ✔	0.05 ✔	0.11 ✔
Small Bowel	6.89 ✘	5.45 ✘	0.88 ✔	4.90 •	7.48 ✘	0.57 ✔
Stomach	17.09 ✘	4.11 •	2.75 •	0.32 ✔	0.30 ✔	0.01 ✔

**Figure 5.**
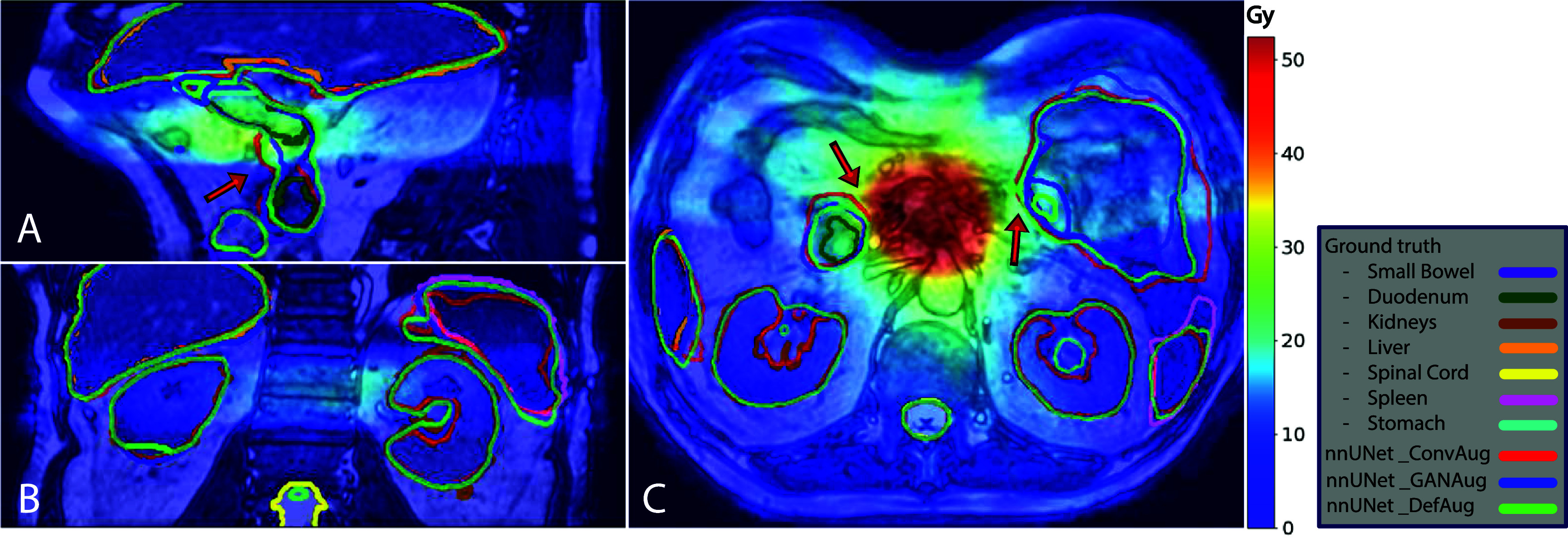
Predictions from various augmentation approaches in sagittal (A), coronal (B), and axial (C) planes, overlaid on a b3DVane image with its corresponding dose map. The dose levels are represented by the color bar on the right in Gy. Red arrows indicate geometric errors in steep dose gradient areas, which significantly impact dose comparison results. The ConvAug approach demonstrates larger errors in these regions, particularly in the duodenum and small bowel.

We also investigated the contours closer to the target to assess how geometric accuracy impacts dose criteria similarity across each organ. Figure 5 illustrates the comparison between the predicted contours superimposed on the dose distribution with the ground-truth contours. Accordingly, geometric discrepancies in regions with steep dose gradients result in more pronounced differences in dose criteria. In other words, small geometric errors in areas closer to the target are penalized more, resulting in greater dissimilarities in dose criteria metrics.

## 4. Discussion

This study demonstrated that nnU-Net performs well with limited data, showing statistically significant superiority compared to the other models we considered. SegResNet outperformed ResU-Net, likely due to the regularization penalty in its decoder path for reconstructing the auto-segmentation result (see section A.1).

To enhance the performance of auto-segmentation models, deformation-based image augmentation methods are typically applied either globally or locally, without considering the spatial information from patient anatomy and internal structures. This approach is inconsistent with observed inter-patient variability and disregards the established correlation between patient size and organ dimensions (Simard *et al* 2003, Javaid *et al* 2019). Kovac *et al* introduced an anatomy-informed data augmentation technique to improve prostate cancer detection in MRI by simulating realistic deformations of the prostate and adjacent organs (2023). However, biomechanical modeling of the pancreas poses challenges due to its heterogeneous tissue, complex interactions with surrounding organs, and substantial inter-patient variability. The computational complexity of these methods and the requirement for high-quality, patient-specific imaging data further complicate accurate applications. In contrast, the proposed sgDefAug approach addresses all these limitations by incorporating the spatial information of OARs when deforming patient anatomy. This method generates more realistic and anatomically consistent augmented images that better reflect the true variability seen across patients by taking into account the positions and sizes of organs within the body, potentially leading to more robust and generalizable models.

Investigation of the dosimetric analysis subsequent to geometric analysis reveals that higher Dice scores and lower ASDs mostly lead to reduced dose discrepancies between predictions and ground truth. This can potentially impact treatment outcomes and the accurate assessment of absorbed dose by OARs. As demonstrated in table 3, the sgDefAug approach, with its superior geometric accuracy, results in lower dose discrepancies (deviations of less than 1%) across each organ compared to the ground truth. However, geometric analysis alone does not serve as a proper indicator of model performance. Figure 5 demonstrates that in regions with steep dose gradients, geometric discrepancies between predicted and ground-truth contours result in larger differences in dose criteria. This discrepancy may be attributed to the presence of high-dose gradients in regions where small errors in contour prediction occur. Such areas of high-dose gradients can amplify the impact of minor contouring inaccuracies on the resulting DVHs. Conversely, for the OARs that are located further from the tumor and show larger geometric errors, these errors are not as impactful from a dosimetric perspective. For instance, this is evident in the left kidney and spleen, where substantial differences in Dice scores are observed, but the dosimetric criteria remain quite similar. This finding emphasizes the necessity of performing dose criteria analysis rather than solely relying on geometric accuracy metrics across the entire organ, particularly when evaluating regions proximal to high dose gradient areas. However, it is also important to note that reducing geometric inaccuracy and thereby dose dissimilarity in high-dose gradient regions can substantially lower the risk of normal tissue toxicity by acquiring high-quality treatment plans from more accurate contours, leading to fewer side effects and better long-term health outcomes for patients. More accurate delineation of OARs closer to the tumor ensures that these structures are not overdosed, optimizing treatment delivery.

To evaluate the dosimetric impact of the auto-segmentation results, we conducted a dose criteria similarity analysis using a previously established treatment plan. The objective was to assess the similarity between dosimetric parameters for predicted and ground-truth OAR contours within the context of a fixed plan. This approach, while suitable for our specific aims, may not fully capture the potential dosimetric variations that could arise from plan optimization based on different contours. Future research could address this limitation by investigating the dosimetric impact of implementing treatment plans for each predicted contour and the ground truth.

Despite the advantages of sgDefAug, figure 1 reveals that this approach did not uniformly improve the accuracy for all structures. This variability in performance can be attributed to the use of compression belts in MR-Linac imaging processes. These belts are employed to restrict tumor displacement caused by breathing motion during RT treatment. They can induce very large changes in patient anatomy, such as displacing the small bowel in or out of the FOV. It’s important to note that while sgDefAug enhances anatomical realism in many cases, it cannot generate contours for structures that are absent from the FOV. Although these deformations improve model robustness by expanding the range of anatomical configurations, we acknowledge that they may not fully represent the true diversity of anatomical variations seen in a real patient population.

Each of the two augmentation approaches has its advantages and disadvantages. The GANAug approach demonstrates the capability to generate realistic images with authentic patient structures, particularly in terms of accurate contrast characteristics. However, this approach requires careful attention, substantial training data, and a more complex process involving the training of multiple AI models. Although it necessitates multiple datasets from different modalities during the initial training phase, once trained, it can be deployed without the need for further refinement. In contrast, the sgDefAug approach, while not generating entirely new patient data, introduces anatomy variations. This characteristic makes it useful across various imaging modalities and image contrasts, provided that spatial information on the OARs is available. Its implementation simplicity and lower computational demands potentially facilitate faster iteration during auto-segmentation model development. The two approaches present distinct trade-offs in terms of implementation complexity and achievable results. Their relative effectiveness may vary across different cancer types and associated OAR contouring tasks, contingent upon factors such as anatomical complexity, input image availability, and image quality. While our study primarily focused on improving auto-contouring model generalizability with limited datasets, we acknowledge that larger datasets could potentially enhance model generalizability and robustness further. Future research directions could explore these approaches and their associated trade-offs across diverse anatomical sites and larger datasets, providing a more comprehensive understanding of their relative advantages in different clinical contexts. Such investigations could help establish practical augmentation strategies for specific clinical scenarios, thereby potentially improving treatment planning accuracy and overall therapeutic efficacy through more precise target delineation.

To the best of our knowledge, this study is the first comprehensive investigation of auto-segmentation of the structures relevant for RT treatment planning from b3DVane images acquired using MR-Linacs for pancreatic cancer patients. The paucity of prior research is likely attributable to the limited availability of suitable high-quality datasets. This underscores the significant challenges of addressing this issue. Several studies have explored automatic contour refinement (ACR) processes and contour modification to enhance the accuracy of deep learning-based auto-segmentation for complex abdominal structures in MRI-guided adaptive RT (Ding *et al* 2022, Sarosiek *et al* 2023, Zarenia *et al* 2023). Ding *et al* employed ACR to refine contours using a dataset of 80 patients, with 65 images acquired from a 3 T MR simulator (MR-SIM), which provides higher soft tissue contrast compared to the Unity MR-Linac. However, the ACR approach is only applicable when an initial set of contours is available for refinement. Furthermore, the quality of the final output is dependent on the accuracy of the initial input contours; highly inaccurate initial contours may pose significant challenges to the model’s refinement process. Despite these limitations, our model demonstrates superior performance without requiring initial contours in terms of Dice score (0.49–0.79) and ASD (3.29–9.78 mm). This is particularly noteworthy given the constraints of our dataset and the inherent challenges of b3DVane imaging in MR-Linac systems. Recent studies have explored the potential of generating automated contours based on patient-specific characteristics. In these approaches, the model is trained on the images from the first session for each patient, and then the trained model is applied to other session images to generate contours (Li *et al* 2022). However, these methods necessitate multiple training processes and require a set of manual contours on the first session image as a prerequisite. Alternative research has focused on auto-segmentation techniques utilizing multi-sequence MR images, which offer the advantage of multiple input sources. Nevertheless, this approach is not entirely applicable in MR-Linac settings and requires additional imaging during the online workflow, where time constraints are significant (Liang *et al* 2020, Amjad *et al* 2022).

Beyond the scope of the MR-Linac treatments, in the context of abdominal MR auto-segmentation, Chen *et al* employed 3 T MR images and a significantly larger patient cohort (10 times greater), along with expert manual contours. Despite these advantages, our results are comparable to their work. They reported OAR Dice scores ranging from 0.80 to 0.96, with an inference time of 1 min, which is approximately twice the duration required by our approach. This comparison suggests that our overall auto-segmentation and data augmentation approach performs comparably to those with higher quality images from a larger patient population (2020).

While our study demonstrates promising results, we focused exclusively on auto-contouring OARs, leaving target delineation to clinicians. This is expected to result in significant time savings when integrated into an online adaptive RT workflow, where time constraints pose a considerable challenge. Our proposed approaches address this issue by facilitating contour generation from preprocessing to postprocessing in under 30 s for the full FOV, rather than confining the process to the PTV+2 cm margin, which is currently employed as a time-saving measure in the online adaptive workflow. This represents a substantial improvement over the 10–20 min partial manual re-contouring process for adaptation while maintaining accuracy. Nevertheless, verification and potential modification of the auto-contours may still be necessary. AI models will inherently have biases and limitations that can lead to errors or suboptimal performance on outlier cases. While our method demonstrates performance comparable to inter-observer variability for specific organs such as the liver (Chlebus *et al* 2019), maintaining clinical oversight and enabling potential refinement of auto-generated contours remains an essential quality assurance measure. However, we firmly believe our approach can serve as a valuable starting point to streamline the contouring workflow, even if complete automation is currently infeasible. By providing initial contours that require minor edits, we can significantly reduce the clinicians’ manual workload and improve efficiency. Furthermore, the growing number of patients associated with radiotherapy will enable continued model refinement and less need for human intervention over time. Future research will investigate the time savings and workflow impact when clinicians review and, where necessary, edit the auto-segmentation results. Quantifying these efficiency gains will be crucial for demonstrating the practical clinical utility of our approach.

Although cycleGANs do not inherently require paired datasets, our initial model training, conducted without any prior registration and deformations using Platipy, failed to converge. To address this, we implemented a registration-based approach, which successfully resolved the convergence issue. This finding underscores the importance of image alignment in facilitating generative model convergence in scenarios with limited data availability. Another constraint in training the cycleGAN was the scarcity of abdominal CT scans with all the required OAR contours. This necessitated the use of pediatric CT scans with expert manual contours as the only suitable dataset. This dataset comprised subjects ranging in age from 5 d to 16 years, with a mean age of 7 years. Access to a dataset with an age distribution more closely aligned with the adult RT patient population could potentially enhance auto-segmentation performance. Regarding our cycleGAN outputs, while they underwent visual validation, the quality of these synthetic images was not critical to the final model performance. This is due to our two-stage strategy of first training the auto-segmentation model using synthetic images generated by the cycleGAN and subsequently fine-tuning the model weights on real images. This strategy allowed the model to adjust its parameters to real image characteristics and mitigate any discrepancies introduced by the synthetic training data, i.e. synthetic artifacts or the presence of unwanted structures.

The model parameters and image specifications were constrained by available computational hardware limitations, and the training outcomes could potentially vary with access to more advanced computational resources. This limitation was particularly pronounced in the GANAug approach, which required training multiple computationally intensive models simultaneously.

## 5. Conclusions

The implementation of advanced augmentation approaches significantly enhances model performance compared to ConvAug methods. This improvement underscores the substantial impact of these approaches on the AI models’ performance and subsequent clinical outcomes. The sgDefAug approach generates more accurate auto-contours that demonstrate closer alignment with ground-truth dose criteria, enabling more precise treatment planning and more accurate OAR dose reporting, even with a limited training dataset which is typically challenging for AI models. The combination of this clinical practicality with computational efficiency and implementation simplicity makes it particularly suitable for integration into the MR-Linac online adaptive workflow. This integration could potentially streamline the current process within the critical pre-treatment window for pancreatic cancer patients, where time constraints often limit the ability to adapt to all anatomical changes. Therefore, the implementation of this approach could potentially improve treatment delivery and patient outcomes.

## Data Availability

The data cannot be made publicly available upon publication due to legal restrictions preventing unrestricted public distribution. The data that support the findings of this study are available upon reasonable request from the authors.

## Appendix

This section demonstrates the configuration and implementation of our deep learning-based models for our project repeatability.

### A.1. Model configuration

Various loss functions were investigated to train the auto-segmentation models, including a Hausdorff distance-based loss function, Dice loss (Ma *et al* 2021), cross-entropy loss (Goodfellow *et al* 2016), and their weighted average (DiceCE). Additionally, a weighting factor was introduced into the loss function to address the class imbalance, assigning greater penalties for misclassification of smaller organs with fewer voxels (Milletari *et al* 2016, Li *et al* 2019). The weighted average of Dice loss and Cross Entropy loss demonstrated superior results compared to the other tested loss functions. Incorporating Hausdorff distance into the loss function did not improve the accuracy. Similarly, the implementation of weighting factors to address class imbalance did not enhance segmentation performance. These evaluations were conducted to identify the optimal loss function for our segmentation models.

Figures A1 and A2 illustrate the architecture design of the implemented SegResNet and ResU-Net. Both models incorporated two convolutions per block in the encoder and decoder paths. The SegResNet model features one encoder and two decoders: one for the segmentation path and the other for regularizing the segmentation output using a variational auto-encoder (VAE). The VAE branch reconstructs the input image, serving as a regularization mechanism for the shared encoder during training. The encoder–decoder structure, coupled with the VAE branch, enables efficient feature extraction and segmentation while maintaining model generalizability through regularization.

Figure A3 illustrates the architecture design and the associated loss functions of the implemented PatchGAN discriminator cycleGAN. The generator architecture includes both encoder and decoder blocks with nine residual blocks in the bottleneck, while the discriminator consists of an encoder path. Since both cycles share the same networks, in total, two generators and two discriminators were trained during the training process.
